# Analysis of the efficacy of photobiomodulation therapy in the treatment of oral lichen planus: A systematic review and meta-analysis of randomized and non-randomized clinical trials

**DOI:** 10.4317/jced.63975

**Published:** 2026-07-29

**Authors:** Lívia Maria Martins Aragão, Gabriella Alves Julião Costa, Janderson Fernando da Silva, Marcela Maria Fontes Borges Franco, Paulo Goberlânio de Barros Silva, Mário Rogério Lima Mota

**Affiliations:** 1 Department of Dental Clinic, Division of Oral Pathology, Faculty of Pharmacy, Dentistry and Nursing, Federal University of Ceará, Fortaleza, Ceará, Brazil; 2 Department of Dentistry, Unichristus, Fortaleza, Ceará, Brazil; 3 Haroldo Juaçaba Hospital, Ceará Cancer Institute, Fortaleza, Ceará, Brazil

## Abstract

**Background:**

Oral lichen planus (OLP) is a chronic autoimmune disease that negatively affects patients' quality of life through symptoms such as pain, irritation, bleeding, and ulceration. Despite numerous therapeutic approaches, there is no definitive cure, and conventional corticosteroid therapy is often associated with prolonged use and adverse effects. Photobiomodulation therapy (PBMT) has emerged as a promising non-invasive alternative with no reported side effects. This systematic review aimed to evaluate the efficacy of PBMT in the treatment of OLP.

**Materials and Methods:**

Electronic searches were conducted in PubMed, LILACS, Livivo, Scopus, Embase, Web of Science, and EBSCO up to March 1, 2025. Randomized and non-randomized clinical trials involving human participants were included, comparing PBMT with conventional pharmacological therapy. Risk of bias was assessed using the Cochrane tools, and the certainty of the evidence was evaluated using the GRADE approach.

**Results:**

A total of 4,118 studies were identified, and 15 met the inclusion criteria after screening. Meta-analysis showed that PBMT slightly reduced pain levels compared with conventional therapy but did not significantly affect disease severity or anxiety scores. However, PBMT was associated with higher rates of complete mucosal healing and lower recurrence rates in patients with erosive OLP.

**Conclusions:**

PBMT appears to be a safe and effective therapeutic alternative for the management of OLP, particularly by reducing pain, promoting mucosal healing, and decreasing lesion recurrence. These findings support its potential role in clinical practice, although further high-quality randomized clinical trials are warranted to strengthen the current evidence.

## Introduction

Oral lichen planus (OLP) is a chronic mucocutaneous inflammatory autoimmune disease of unknown etiology, presenting as reticular white lesions that may or may not be accompanied by atrophic and/or erosive lesions, in plaque or bullous form (1). Studies indicate that OLP is a T-cell-mediated disease, with mechanisms that result in lymphocytic infiltration in the lamina propria and epithelial tissue (2). Mucosal LP is characterized by resistance to treatment and persistent inflammation (3). Corticosteroid therapy is the primary treatment for OLP, and the available therapies seek to reduce the symptoms of the disease. However, chronic use of these medications can cause several adverse effects for the patient, such as muscle weakness, pathological fractures, and anemia, among others (4 , 5). Photobiomodulation therapy (PBMT) is a non-invasive, non-pharmacological option with minimal adverse effects (6). It is beneficial for patients who use medications with adverse reactions or for patients who do not respond to pharmacological treatments. PBMT involves using lasers to influence cellular metabolism, which leads to increased levels of adenosine triphosphate (ATP), improved immunity, and modulation of pro- and anti-inflammatory cytokines and growth factors. Reported benefits include analgesia, increased viability, cell proliferation and migration, modulation of inflammation, and improved tissue repair (6 , 7). Studies addressing PBMT as part of OLP management show varied results, since parameters that allow for reliable analysis of this tool have not yet been proposed, in addition to the risk of Bias observed in the literature. Some clinical trials (6 , 8) have shown that PBMT had therapeutic effects similar to topical corticosteroid therapy with clobetasol propionate, which indicates an alternative to conventional pharmacological treatment. According to the WHO, OLP is a potentially malignant disorder (9). However, OLP has one of the lowest rates of malignancy among potentially malignant oral disorders (PMOD), ranging from 0.44% to 2.28% of cases (10). One hypothesis for this is that there is high activity of tumor suppressor genes, especially the p53 gene (10). Despite the lack of evidence on the oncological safety of using PBMT in POMDs, due to a hypothetical acceleration of the malignant transformation of these lesions, there are no reports in the current literature on the malignant transformation of OLP after treatment with PBMT. In this sense, the present systematic review seeks to analyze the therapeutic potential of photobiomodulation therapy for the management of OLP lesions, with and without association with conventional therapies.

## Materials and Methods

1. Protocol This study was registered in the Prospective International Register of Systematic Reviews (PROSPERO; https://www.crd.york.ac.uk/PROSPERO), under registration code CRD42024574131, and was included in the Preferred Reporting Items for Systematic Reviews and Meta-Analyses (PRISMA) checklist (11). Institutional Review Board approval was not required for this study, as it is based exclusively on previously published data. 2. Search strategy A systematic review was conducted to answer the following question: "In patients with oral lichen planus, is photobiomodulation therapy alone or in combination with pharmacological treatment effective?" using the PICOS strategy: Population (P): Patients with symptomatic oral lichen planus, with or without associated skin lesions. Intervention (I): Photobiomodulation therapy. Control (C): Patients treated with conventional drug therapy. Outcome (O): Clinical improvement of lichen planus lesions. Study design (S): Randomized and non-randomized clinical trials. A specific search strategy was developed for each database using the descriptors "Oral Lichen Planus," "Lichen Planus," and "Low-Level Light Therapy." Appropriate truncations and word combinations were selected and adapted for each database search (Supplementary Material 2). 3. Inclusion criteria Randomized and non-randomized clinical trials evaluating the therapeutic potential of photobiomodulation therapy in the treatment of symptomatic oral lichen planus; studies conducted in humans without restriction of age, gender, ethnicity, publication date, or language; studies with intervention groups (photobiomodulation therapy) and control groups (conventional drug therapy). 4. Exclusion criteria Case report studies, systematic reviews, studies that did not present groups for comparison of results (photobiomodulation therapy), duplicates, and/or studies that did not report results after the end of the research; articles whose description of research follow-up data was incomplete or with inadequately described outcomes; articles that did not detail the diagnostic process for oral lichen planus. 5. Sources of information The search was performed in Medline via PubMed, Lilacs, Livivo, Scopus, Embase, Web of Science, and EBSCO. The gray literature was investigated, and Open Gray, Google Scholar, and ProQuest were included. A manual search was also performed in the references of the selected articles. The search included all articles published on or before June 4, 2024, with no time restrictions. The search was updated on March 1, 2025. 6. Study selection The selection was performed in two phases. In phase 1, two reviewers (JFS and LMMA) independently reviewed the titles and abstracts of all citations in the electronic database. Phase 1 was conducted using a web application for systematic reviews (Rayyan®, Qatar Computing Research Institute, Doha, Qatar) (12). Articles that did not meet the inclusion criteria were excluded. In phase 2, the same reviewers independently applied the inclusion criteria to the full texts of the articles. One reviewer (MMFBF) critically appraised the reference lists of the selected studies. Any disagreements were resolved when the two authors reached an agreement. When they did not reach consensus, the third and fourth authors (MMFBF and GAJC) participated in the final decision. The researcher, PGBS, performed the statistical analysis. 7. Data collection process One researcher (PGBS) extracted the data from the selected studies, and another (MMFBF) verified all the information obtained. Any disagreement between the two authors was discussed until a consensus was reached. A third author (MRLM) made the final decision if the two authors could not reach an agreement. 8. Variables The study variables were the following complications: painful symptoms, recurrence of OLP lesions, and quality of life scores. 9. Assessment of risk of Bias and study quality Risk of Bias was assessed independently by two authors (MMFB and GAJC). The revised Cochrane risk of bias tool for randomized clinical trials (RoB-2) was used to assess RoB in randomized clinical trials (RCTs) (13), and the Cochrane Risk of Bias In Non-Randomized Studies of Interventions (ROBINS-I) method was used to assess RoB in non-randomized clinical trials (n-RCTs) (13). In addition, RevMan Software (Review Manager, version 5.3, Cochrane Collaboration, Copenhagen, Denmark) generated the RoB summary figures. 10. Meta-analysis For the meta-analysis, means, standard deviations, and numbers of participants were extracted from studies across various evaluation periods. The difference in means was used for pain scales (VAS 0-10); for VAS scales ranging from 0-100, these were converted to 0-10. For disease severity and disease-related anxiety scales, the difference in standardized means was used to calculate Cohen's d, as they are different scales. For the incidence of total mucosal healing and disease recurrence, the combined relative risk was calculated. In all approaches, the inverse variance method with random effects was used. Heterogeneity was calculated using the I² coefficient. A one-out analysis was performed by removing each study individually to verify its weight in the meta-analysis. A funnel plot was constructed, and Egger's and Begg's tests were performed to analyze the risk of publication bias. 11. Quality of scientific evidence The quality of evidence was assessed using the Grading of Recommendations, Assessment, Development, and Evaluation (GRADE) approach, which reflects the reliability of the estimated effect of the item evaluated. The GRADE profile obtained certainty evidence using the free online software GRADE pro-GDT, available at http://gdt.guidelinedevelopment.org, which was downgraded or upgraded according to the importance of certain aspects (e.g., study design, bias, consistency, frankness, heterogeneity, precision, publication bias, and others identified in the included studies) (14).

## Results

**Figure 1:**
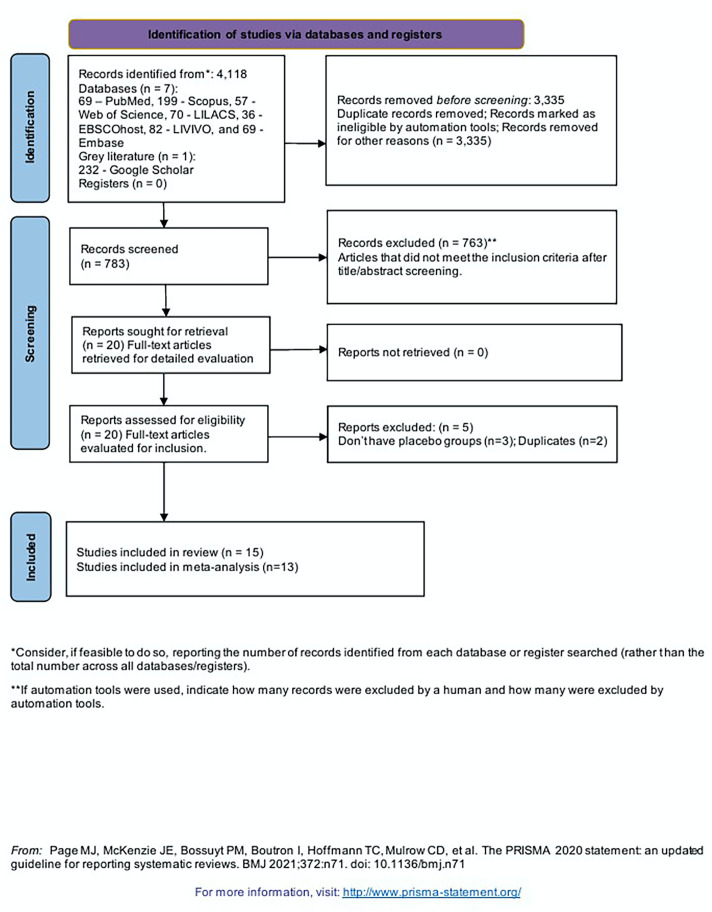
PRISMA 2020 flow diagram for new systematic reviews which included searches of databases and registers only.

12. Article screening and inclusion flow A total of 4,118 articles were reviewed, and 3,335 duplicates were removed after reading the titles and abstracts and excluding those that did not fit the PICOS strategy. After reading the articles in full, 20 remained. Three articles were excluded due to the absence of placebo groups, and two others were removed as duplicates (dissertation/thesis vs. article). Therefore, 15 articles were included in the systematic review (Fig. 1).

1

13. Descriptive analysis The 15 studies included in this systematic review analyzed different therapeutic approaches for oral lichen planus, totaling 890 patients. All studies demonstrated a good distribution of participants between the test and control groups. Only five studies did not report the gender of participants (Ai-Ping et al., 2016; El Shenawy & Eldin, 2015; Mohamed et al., 2024; Othman et al., 2016; Panchal et al., 2023) (15 - 19). Overall, there was a balanced distribution between men and women, with a slight female predominance. Regarding age, most studies included patients with a mean age ranging from 30 to 60 years (15 - 19) (Table 1).

Table 1

Ten studies used independent group designs (Ai-Ping et al., 2016; Dillenburg et al., 2014; Ferri et al., 2021; Jain et al., 2021; Kazancioglu et al., 2015; Laxmi et al., 2015; Matsumoto et al., 2019; Mirza et al., 2018; Mohamed et al., 2024; Panchal et al., 2023) (15 , 17 , 20 - 25), while five adopted a crossover design (Jajarm et al., 2011; El Shenawy & Eldin, 2015; Othman et al., 2016; Salinas-Gilabert et al., 2022; Sanjay et al., 2022) (4 , 16 , 18 , 26 , 27) (Table 1). The therapeutic modalities evaluated included photodynamic therapy, photobiomodulation, low-level laser therapy, CO2 laser therapy, and topical corticosteroids. The most frequently used corticosteroid was 0.1% triamcinolone acetonide, reported in six studies (Dillenburg et al., 2014; Jain et al., 2021; Laxmi et al., 2015; Mohamed et al., 2024; Salinas-Gilabert et al., 2022; Sanjay et al., 2022) (4 , 17 , 20 , 21 , 23 , 27). Additionally, five studies assessed the combination of laser therapy and corticosteroids (Jain et al., 2021; Laxmi et al., 2015; Othman et al., 2016; Panchal et al., 2023; Sanjay et al., 2022) (18 , 19 , 21 , 23 , 27), and three studies compared low-level laser therapy and photodynamic therapy with corticosteroid treatment (Mirza et al., 2018; Salinas-Gilabert et al., 2022; Sanjay et al., 2022) (4 , 25 , 27) (Table 1). Treatment duration varied across studies, with protocols lasting from four to twelve weeks. The frequency of laser sessions ranged from twice a week to once every two weeks. The results analyzed included improvement in healing and recurrence. Most studies reported significant improvement with the use of photobiomodulation therapy and CO2 laser compared to the use of corticosteroids alone (Table 2).

Table 2

**Figure 2:**
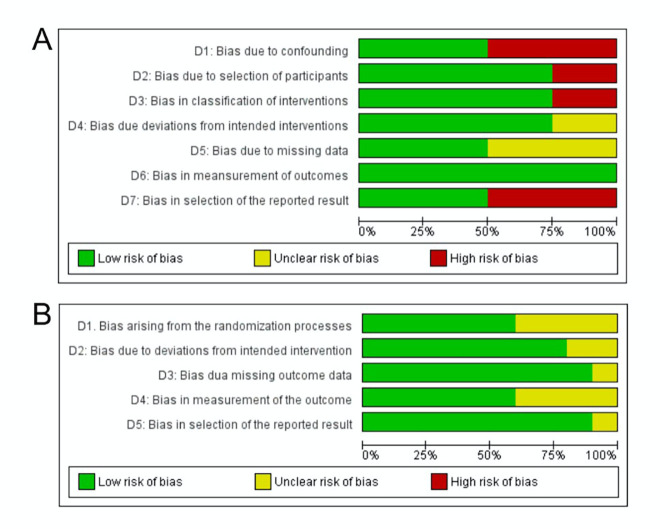
(A) Analysis of the risk of Bias in non-randomized clinical trials; (B) Analysis of the risk of Bias in randomized clinical trials.

14. Risk of Bias Non-randomized clinical trials presented a risk of Bias ranging from high to low. The ROBBIN domains, specifically Bias due to confounding and Bias in the selection of reported results, presented the worst performances, with half of the articles showing a high risk of Bias. The domains Bias due to selection of participants and Bias in classification of interventions presented a quarter of the articles with a high risk of Bias, and the domains Bias due to deviations from intended interventions and Bias due to missing data presented 25% and 50% of the articles with a moderate risk of Bias (Fig. 2A).

2

**Figure 3:**
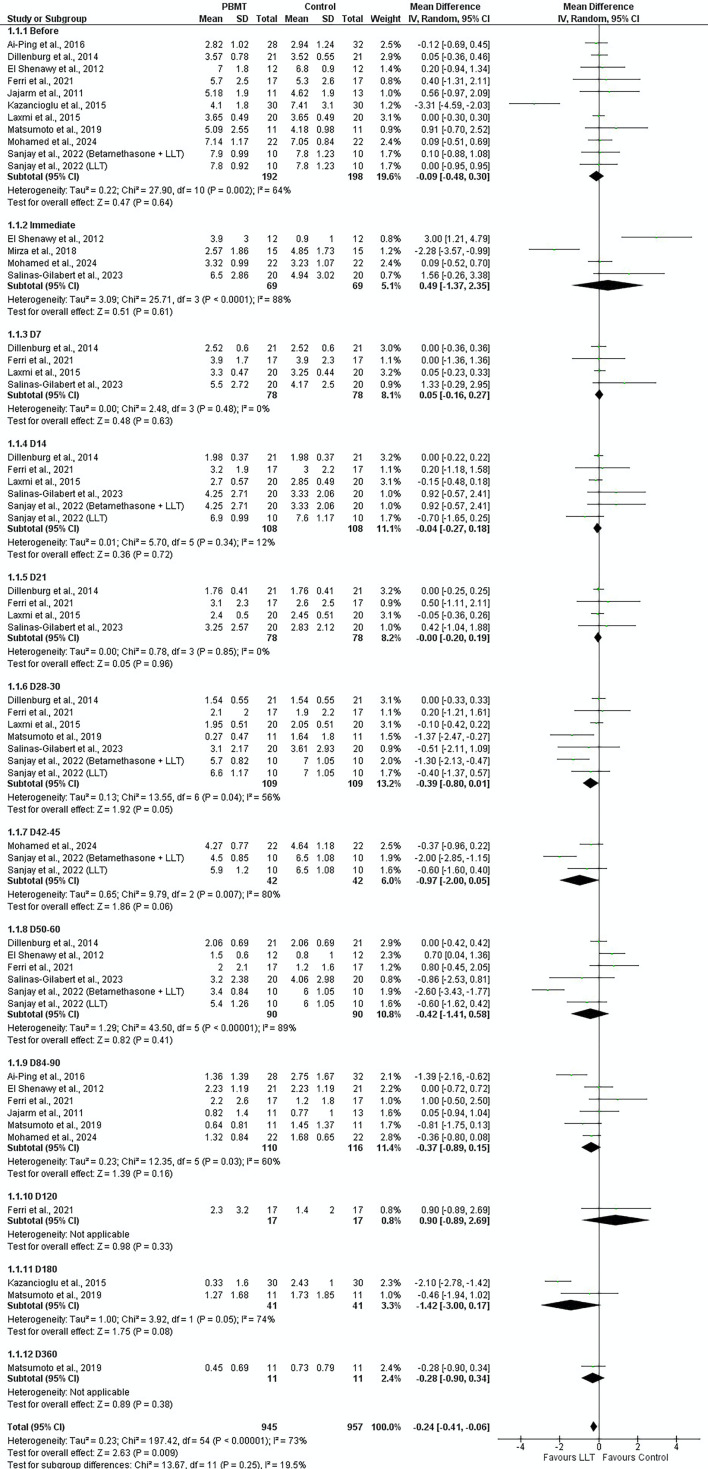
Forest plot of the included studies assessing pain using the VAS scale.

Randomized clinical trials presented a predominantly low risk of Bias. The domains Bias arising from the randomization processes and Bias in measurement of the outcome presented 40% of the articles with intermediate risk. All other domains presented more than 80% of the articles with low risk of Bias (Fig. 2B). 15. Meta-analysis: PBMT slightly reduces pain in patients with erosive lichen planus compared to conventional treatment. After applying the inclusion and exclusion criteria, of the 15 studies included in the systematic review, 13 studies were included in the meta-analysis, and it was possible to evaluate five outcomes (Fig. 3).

3

**Figure 4:**
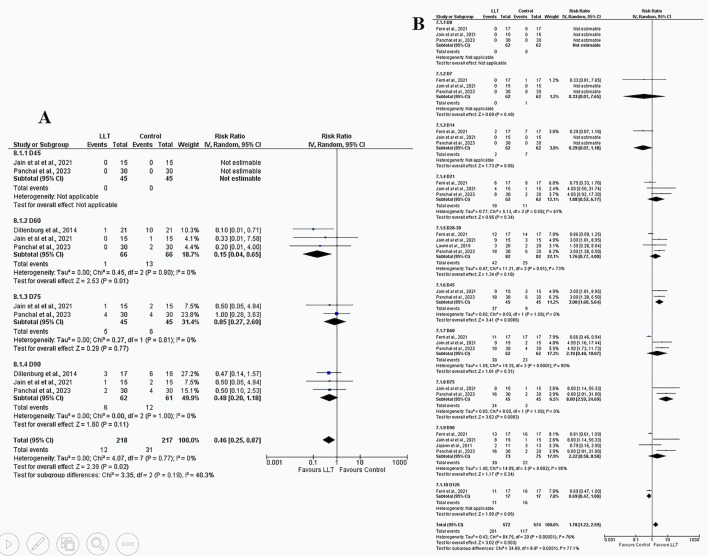
Forest plots of primary and secondary outcomes.(A) Recurrence rate of lesions (secondary outcome). (B) Healing response of lesions (secondary outcome).

Regarding pain control, all studies used the visual pain scale. However, the evaluation period ranged from before to immediately after PBMT to one year after the start of treatment, for a total of 1,902 evaluations. There was no significant difference in the assessment of pain scores before treatment (p=0.640), there was moderate significant heterogeneity (p=0.002, I² = 64%), and the one-of-out analysis showed no change in the outcome after individual exclusion of each study (Fig. 3). Immediately after the start of treatment, there was also no significant improvement when the two groups were compared (p=0.610), there was high heterogeneity (p=0.001, I² = 88%), and the one-out analysis showed no change in the outcome after individual exclusion of each study (Fig. 3). After 7 (p=0.630), 14 (p=0.720), and 21 (p=0.960) days of treatment initiation, there was no significant improvement when the two groups were compared, there was no significant heterogeneity (p=0.630, p=0.340, and p=0.850, respectively), and the one-out analysis showed no change in the outcome after individual exclusion of each study (Fig. 3). After 28-30 days (p=0.050) and 42-45 days (p=0.060) of treatment, there was a slight reduction in pain scores in the PBMT group of -0.39 and -0.97 points, with no significant difference between the groups. Heterogeneity was significant (p=0.040, I² = 56%; and p=0.007, I² = 80%) in both periods, and the one-of-out analysis showed that the removal of the studies by Dillenburg et al., 2014, and Ferri et al., 2021, significantly benefited t h e control group with a reduction of -0.57 [95% CI = -1.13, -0.01] and -0.45 [95% CI = -0.88, -0.02] pain points in the 28-30 day period after the start of treatment, but it was not possible to perform this analysis after 42-45 days because only two studies were included (Fig. 3). After 50-60 days (p=0.410) and 84-90 days (p=0.160) of treatment, there was no significant improvement when the two groups were compared, there was significant heterogeneity (p=0.001, I² = 89%; and p=0.030, I² = 60%, respectively), and the one-of-out analysis showed no change in the outcome after individual exclusion of each study (Fig. 3). After 120 days, only Ferri et al. (2021) evaluated pain, finding no significant benefit in the use of PBMT (p=0.330). After 180 days, only Kazancioglu et al. (2015) and Matsumoto et al. (2019) evaluated pain, finding the same outcome. Similarly, after 360 days, Matsumoto et al. (2019) evaluated pain, finding no significant benefit in the use of PBMT (p=0.380) (Fig. 3). When all periods were evaluated together, a significant reduction (p=0.009) of -0.24 [95% CI = -0.41, -0.06] points in the pain score in the PBMT group, with significant heterogeneity (p=0.001, I² = 73%) and no significant differences between the evaluation periods (p=0.250) (Fig. 3). The Egger test (p=0.210) showed no significant risk of publication bias (Supplement 2) (http://www.medicinaoral.com/medoralfree01/aop/jced_63975_s02). 1.16. Meta-analysis: PBMT does not reduce the severity of lichen planus or anxiety scores associated with the disease compared to conventional treatment About the severity of lichen planus, the scales used were quite different, but all scales were ascending, leading to the evaluation of 1533 events through standardized mean differences. There was no significant difference in the evaluation of severity scores before treatment (p=0.990), after seven (p=0.510), 14 (p=0.750), 21 (p=0.180), 28-30 (p=0.470), 42-60 (p=0.740), or 84-90 days (p=0.650) after treatment. There was significant heterogeneity only in the period 28-30 days after the start of treatment (p=0.002, I² = 84%), and it was possible to perform a one-of-out analysis in the pre-treatment and 84-90 days after therapy periods, which had more than two studies with data available for analysis. In both cases, the one-of-out analysis showed no change in the outcome after individual exclusion of each study (Supplement 1) (http://www.medicinaoral.com/medoralfree01/aop/jced_63975_s01). In the period after 120-180 days, the PBMT group had slightly higher mean scores than the control groups (p=0.002), with a Cohen's D of +0.47 [95% CI = 0.17, 0.77], there was no significant heterogeneity (p=0.630), and it was not possible to perform a one-out analysis because only two studies were included (Supplement 1) (http://www.medicinaoral.com/medoralfree01/aop/jced_63975_s01). When all periods were evaluated together, no significant reduction in lichen planus scores was observed (p=0.066); there was significant heterogeneity (p=0.003, I² = 51%), and no significant differences between the evaluation periods (p=0.080) (Supplement 1) (http://www.medicinaoral.com/medoralfree01/aop/jced_63975_s01). The Egger test (p=0.375) showed no significant risk of publication bias (Supplement 2) (http://www.medicinaoral.com/medoralfree01/aop/jced_63975_s02). Similarly, different anxiety scales were used, but all scales were ascending, leading to the evaluation of 414 events through standardized mean differences. There was no significant difference in the evaluation of anxiety scores before treatment (p=0.150), after seven (p=0.860), 14 (p=0.900), 21 (p=0.340), 30 (p=0.510), 60 (p=0.630), or 90 (p=0.050) days after treatment. There was significant heterogeneity only in the period after 60 days from the start of treatment (p=0.008, I² = 86%), and it was not possible to perform this one-of-out analysis in any period, as none had more than two studies with data available for analysis (Supplement 1) (http://www.medicinaoral.com/medoralfree01/aop/jced_63975_s01). When all periods were evaluated together, no significant reduction in anxiety scores was observed (p=0.406), and there was significant heterogeneity (p=0.030, I² = 50%) or significant differences between the periods of evaluation (p=0.290) (Fig. 4).

4

Egger's test (p=0.042) demonstrated a significant risk of publication bias (Supplement 2) (http://www.medicinaoral.com/medoralfree01/aop/jced_63975_s02). 1.17. Meta-analysis: PBMT increases the incidence of total mucosal healing and decreases the recurrence rate of lichen planus compared to conventional treatment Regarding the incidence of mucosal healing, 1146 events could be evaluated. There was no significant difference before treatment (p=1.000), after seven (p=0.490), 14 (p=0.080), 21 (p=0.340), and 28-30 (p=0.180) days after treatment. There was significant heterogeneity only in the period after 28-30 days from the start of treatment (p=0.010, I² = 61%) and when the one-of-out analysis was performed on these, the removal of the study by Ferri et al., 2021 increased the incidence of total mucosal healing by 4.00 [95% CI = 1.21, 13.22] times the incidence of total mucosal healing (p=0.020) after 21 days of treatment and 2.75 [95% CI = 1.52, 4.97] after 28-30 days of treatment (p=0.008) compared to the control (Fig. 4). After 45 days, the incidence of mucosal healing was 3.00 [95% CI = 1.60, 5.64] times higher in the PBMT group (p=0.006), there was no significant heterogeneity (p=1.000), and it was not possible to perform a one-of-out analysis, as only two studies had data available for this evaluation period (Fig. 4). After 60 days of treatment, there was no significant difference between the PBMT and control groups (p=0.310). There was no significant heterogeneity (p=1.000). Still, in the one-of-out analysis, the removal of the study by Ferri et al., 2021 increased the incidence of complete mucosal healing by 4.50 [95% CI = 2.06, 9.84] times the incidence of total mucosal healing (p=0.002) (Supplement 2) (http://www.medicinaoral.com/medoralfree01/aop/jced_63975_s02). After 75 days, the incidence of mucosal healing was 8.00 [95% CI = 2.59, 24.69] times higher in the PBMT group (p=0.003). There was no significant heterogeneity (p=1.000), and it was not possible to perform a one-out-of analysis, as only two studies had data available for this evaluation period (Figure 6 - Supplementary file 1). After 90 days of treatment, there was no significant difference between the PBMT and control groups (p=0.240), there was no significant heterogeneity (p=1.000), and the one-of-out analysis showed no change in the outcome after individual exclusion of each study. After 120 days of treatment, only Ferri et al. (2021 had data available, showing no significant clinical benefit in the use of PBMT (p=0.050) (Fig. 4). When all periods were evaluated together, there was a 1.78 [95% CI = 1.22, 2.59] times increase in the incidence of total mucosal healing when PBMT was used (p=0.003), there was significant heterogeneity (p=0.003, I² = 76%) and significant differences between the evaluation periods (p=0.001), with the best performances occurring between the 21-90 day periods (p=0.001) (Figure 6). The Beggs test (p=0.691) showed no significant risk of publication bias (Supplement 2) (http://www.medicinaoral.com/medoralfree01/aop/jced_63975_s02). Regarding the incidence of disease recurrence, 435 events could be evaluated. There was no significant difference before treatment (p=1.000) or after 75 (p=0.70) or 90 (p=0.110) days after treatment, but after 60 days of treatment (p=0.010), there was a 0.15 [95% CI = 0.04, 0.65] times in the risk of recurrence in the PBMT groups compared to the control. There was no significant heterogeneity in any period. When the one-of-out analysis was performed in periods with more than two studies, the removal of the study by Dillenburg et al., 2014, significantly diluted the outcome observed in the 60 days (p=0.022) (Fig. 4). When all periods were evaluated together, there was a 0.46 [95% CI = 0.25, 0.87] reduction in the incidence of disease recurrence when PBMT was used (p=0.020), with no significant heterogeneity (p=0.770) or significant differences between the evaluation periods (p=190) (Supplement 3) (http://www.medicinaoral.com/medoralfree01/aop/jced_63975_s03).. Beggs' test (p=0.48) showed no significant risk of publication bias (Supplement 2) (http://www.medicinaoral.com/medoralfree01/aop/jced_63975_s02). 1.18. Certainty of evidence The quality of evidence was assessed using the GRADE (Grading of Recommendations, Assessment, Development, and Evaluation) methodology, which indicates the degree of confidence in the estimates of observed effects. The GRADE profile was constructed with the aid of the free GRADEpro GDT software (http://gdt.guidelinedevelopment.org), considering criteria such as the type of study design, risk of Bias, consistency between results, direction of effect, variability (heterogeneity), precision of estimates, possibility of publication bias, and other relevant factors identified in the included studies (Table 3).

Table 3

Photobiomodulation showed better results than pharmacological intervention in reducing recurrence (high certainty) and pain (moderate certainty). It also favored healing, although with low certainty due to high heterogeneity. For anxiety and clinical scores, there was no significant difference, with low certainty evidence. These findings highlight the potential of photobiomodulation in critical outcomes.

## Discussion

This systematic review included 15 clinical trials, comprising 11 randomized and four non-randomized studies, which analyzed the potential therapeutic effects of photobiomodulation for managing OLP lesions. This review provides an updated and comprehensive synthesis of the clinical evidence on the use of PBMT for LPO, incorporating recent trials and offering a broader analysis compared to previous reviews. As a result, we found that PBMT had three main positive biological effects in the treatment of OLP, where it reduced painful symptoms, increased the total healing rate of the mucosa, and reduced the recurrence rate of lesions. Patients undergoing OLP treatment with PBMT showed a slight reduction in painful symptoms, as did patients who underwent treatment with a drug, which corroborates the findings in the literature. Soliman et al. 2025 compared the therapeutic effect of PBMT alone to the effect of 0.5% triamcinolone acetonide and concluded that both therapies similarly reduced pain scores. However, the authors highlighted laser therapy as a therapeutic modality free of adverse effects, unlike the pharmacological treatment, which in some cases led to mucosal atrophy, secondary candidiasis, adrenal insufficiency, and gastrointestinal disorders. In this meta-analysis, it was possible to observe that PBMT demonstrated a significant reduction in pain in the short term (D15), with a relative risk (RR) of 0.15 (95% CI: 0.04-0.65; p = 0.01), favoring the intervention. However, this superiority was not observed in the longer term (D75: RR = 0.85; 95% CI: 0.27-2.66; D90: RR = 0.24; 95% CI: 0.02-2.18), suggesting a possible more significant therapeutic effect at the beginning of treatment. The overall analysis of the studies showed a significant reduction in the risk of pain (RR = 0.46; 95% CI: 0.25-0.87), with no heterogeneity (I² = 0%). The visual analog scale (VAS) used in the studies was considerably homogeneous, which facilitates comparison, unlike other variables such as severity and anxiety, which did not show significant results with PBMT. The results corroborate the findings of Nammour et al. (28), who also observed that PBMT and corticosteroid therapy similarly reduced pain rates. Both treatments act by reducing cytokines and pain mediators, such as prostaglandins, which are reduced in both treatments. This may cause similar analgesic effects in both groups (10). This data indicates the possibility of using PBMT as an alternative to corticosteroid therapy. In addition, less painful symptoms indicate an improvement in the patient's oral health-related quality of life (OHRLQ) (29). Daume et al. (29) observed that patients with the erosive-ulcerative form of the disease have significantly reduced OHRQoL indices, which the authors related to the fact that the disease presents itself more aggressively, causing greater pain to affected patients, as well as discomfort and restrictions in eating, speaking, swallowing, and oral hygiene. Parlatescu et al. (30) observed that physical pain was one of the factors that most negatively impacted the quality of life of patients with OLP. Other clinical trials (31 , 32) analyzed OHRQoL in OLP and corroborate the impact of painful symptoms on patients' quality of life. The authors also describe that social and psychological factors significantly affect the quality of life of these patients. The studies analyzed showed a reduction in the recurrence rate of lesions with the use of PBMT. According to Sangar et al. (33), OLP has a high recurrence rate, reaching 54.2% of patients. The authors related the recurrence of the disease to whether or not the patient was a smoker, describing that the recurrence rate of patients with a history of smoking is lower than that of non-smokers. However, this aspect was not evaluated in the present systematic review, since the articles analyzed did not provide this information. Nammour et al. (28) observed similar recurrence rates between the corticosteroid and PBMT groups, with no statistically significant differences. After 12 months, the recurrence rate was 79% and 87.5% for corticosteroids and PBMT, respectively. The authors also reported even better results with photodynamic therapy (aPDT) using 5-aminolevulinic acid (5-ALA) compared to the same therapy with methylene blue. Cafaro et al. treated 82 lesions in 30 patients, and 64 of these lesions achieved clinical resolution. After more than 26 months, 15 patients had no new lesions (34). The reduction in recurrence rate observed can be explained by the theory that PBMT promotes tissue repair by stimulating mitochondrial cytochrome C oxidase activity, which increases ATP production and cellular energy metabolism. This stimulation promotes cell proliferation, collagen synthesis, and angiogenesis, resulting in more efficient tissue repair and regeneration (17 , 35). Thus, compared to corticosteroids, which act predominantly by suppressing various components of the inflammatory process, PBMT appears to activate cellular repair pathways that restore the typical structure and function of the mucosa, thereby reducing the chances of lesion recurrence (17 , 28). Finally, the study observed a benefit of PBMT in increasing the healing rates of erosive OLP lesions. The rates of healing or clinical resolution in symptomatic OLP lesions vary and depend on the extent of the lesion. Le Gatt et al. (36) observed that erosive OLP has the potential for more extensive lesions, which require long-term therapies. Moreover, according to Rivas-Tolosa et al. (37), the erosive nature of these lesions commonly causes pain and can be a potential site for secondary infections, thereby hindering the healing process (38). Studies such as that by Roca et al. (35) reported that PBMT presents clinical improvement for OLP, since this therapy is capable of delaying cell differentiation, improving healing and re-epithelialization, reducing inflammation through immunomodulation, and exerting an analgesic effect. The authors mention that, although more studies are needed and sometimes contradictory results are obtained, recent evidence suggests that PBMT may be as effective as topical corticosteroids, with the benefit of not presenting adverse effects, which makes it a promising therapy. The recommended treatment for erosive OLP lesions is based on the prescription of topical corticosteroids; however, this type of medication may not be the best approach, as remission of the disease is rare and systemic therapies are often necessary (35 - 38). Chronic use of oral corticosteroids is associated with several complications, such as gastrointestinal bleeding, myocardial infarction, heart failure, cerebrovascular events, diabetes mellitus, psychiatric disorders, ophthalmological changes, among other conditions (39). In addition, Pérez-Sayáns et al. (40) identified the direct participation of Bcl-2 and Ki-67 proteins in the pathogenesis of OLP; these proteins are characterized as anti-apoptotic and proliferative markers, respectively. In this context, Mutafchieva et al. (41) observed that PBMT increased the expression of these proteins, suggesting a potential ability to stimulate tissue regeneration in OLP lesions. Complementing these findings, Gambino et al. (42) conducted a comparative study between the effects of PBMT and topical corticosteroid therapy with clobetasol propionate, revealing that PBMT can promote long-term structural tissue changes and positive biological effects. This review has some limitations; the included studies showed heterogeneity in study design, sample size, treatment protocols, and outcome measures, which may limit comparisons. Another relevant point to consider is the lack of standardized protocols for PBMT, and the incomplete presentation of results may introduce bias. Therefore, the findings should be interpreted with caution.

## Conclusions

The present systematic review suggests that PBMT may represent a promising alternative for the treatment of OLP, as it may be effective in reducing pain symptoms, improving healing, and lowering lesion recurrence rates. It also appears to have a favorable safety profile, with fewer reported adverse effects, supporting its potential clinical use. However, these findings should be interpreted with caution due to heterogeneity among the studies and variability in treatment protocols. Further standardized, high-quality studies are needed to establish a consistent PBMT application protocol and to allow more reliable comparisons with corticosteroid therapy.

## Figures and Tables

**Table 1:** Characteristics of the included studies.

Study	Country	Sample (Int/Con)	Intervention	Control	Diagnosis	Protocol
Ai-Ping et al., 2016	China	28/32	PBM	White peony glycosides	Clinical	3×/week, 3 weeks
Dillenburg et al., 2014	Brazil	21/21	PBM	Clobetasol 0.05%	WHO histological	3×/week, 30 days
El Shenawy & Eldin, 2015	Egypt	12/12	PBM	Triamcinolone	WHO-based	2×/week, 2 months
Ferri et al., 2021	Brazil	17/17	PBM + placebo	Clobetasol 0.05%	WHO	2×/week, 8 sessions
Jain et al., 2021	India	15/15	PBM + triamcinolone	Triamcinolone 0.1%	Clinical + histopathology	2×/week, 9 sessions
Jajarm et al., 2011	Iran	11/13	PBM	Dexamethasone	Biopsy-proven OLP	2×/week, 10 sessions
Kazancioglu et al., 2015	Turkey	30/30	PBM	Dexamethasone	Clinical + histological	2×/week, 10 sessions
Laxmi et al., 2015	India	20/20	PBM	Triamcinolone acetonide	WHO-based	2×/week, 5–6 sessions
Matsumoto et al., 2019	Japan	11/7	PBM	Corticosteroids	WHO-based	Variable
Mirza et al., 2018	Saudi Arabia	15/15	PBM	Dexamethasone	Histological	2×/week, 1 month
Mohamed et al., 2024	Egypt	22/22	PBM	Triamcinolone acetonide	WHO-based	2×/week, 5 weeks
Othman et al., 2016	Egypt	12/12	PBM	Corticosteroids	WHO-based	2×/week, max 10 sessions
Panchal, 2023	India	30/30	PBM + triamcinolone	Triamcinolone 0.1%	Histological	2×/week, 9 sessions
Salinas-Gilabert et al., 2023	Switzerland	20/19	PBM + orabase	Triamcinolone 0.1%	Van der Meij & van der Waal	1×/week, 4 sessions
Sanjay et al., 2022	India	10/10/10	PBM	PBM + triamcinolone	Modified WHO	Every 3 days, 5 sessions

1

**Table 2:** Laser parameters of the included studies.

Study	Laser type	λ (nm)	Power	Dose/Energy	Notes
Ai-Ping et al., 2016	Nd:YAG	650	5 mW	250 mJ/pulse	-
Dillenburg et al., 2014	Diode	660	40 mW	6 J/cm²	6 s exposure
El Shenawy & Eldin, 2015	Diode	970	3 W	NR	-
Ferri et al., 2021	Diode	660	100 mW	177 J/cm²	-
Jain et al., 2021	Diode	810	3 W	NR	-
Jajarm et al., 2011	Diode	630	10 mW	1.5 J/cm²	2.5 min
Kazancioglu et al., 2015	Diode	808	0.1 W	120 J/cm²	-
Laxmi et al., 2015	GaAlAs	980	0.8–0.9 W	6 J/cm²	-
Matsumoto et al., 2019	CO₂	-	-	89–268 J/mm²	60–180 s
Mirza et al., 2018	Diode	630	10 mW	1.5 J/cm²	-
Mohamed et al., 2024	Diode	980	300 mW	NR	4 s
Othman et al., 2016	Diode	970	2 W	NR	-
Panchal, 2023	Diode	810	3 W	NR	-
Salinas-Gilabert et al., 2023	NR	-	-	6 J/cm²	30 s
Sanjay et al., 2022	GaAs	904	NR	NR	-

2

**Table 3:** Summary of Findings. Question: Photobiomodulation compared to pharmacological intervention for patients with symptomatic oral lichen planus with or without associated skin lesions.

Certainty Assessment								Nº of patients		Effect		Certainty	Importance
Outcome	No. ofstudies	Studydesign	Risk ofbias	Inconsistency	Indirectness	Imprecision	Otherconsiderations	Photobiomodulation	Pharmacologicalintervention	Relative effect(95% CI)	Absolute effect(95% CI)		
Recurrence	3	Randomizedtrials	Not serious	Not serious	Not serious	Not serious	None	12/218 (5.5%)	31/217 (14.3%)	RR 0.46(0.25 to 0.87)	77 fewer per 1000(from 107 fewer to 19 fewer)	⊕⊕⊕⊕High	CRITICAL
Healing	5	Randomizedtrials	Not serious	Very seriousᵃ	Not serious	Not serious	None	201/572 (35.1%)	117/574 (20.4%)	RR 1.78(1.22 to 2.59)	159 more per 1000(from 45 more to 324 more)	⊕⊕○○Low	CRITICAL
Anxiety	2	Randomizedtrials	Not serious	Seriousᵇ	Not serious	Seriousᶜ	None	-/207	-/207	SMD -0.12(-0.40 to 0.16)	Not estimable	⊕⊕○○Low	IMPORTANT
Lichen Planus Scores	7	Randomizedtrials	Not serious	Seriousᵈ	Not serious	Seriousᵉ	None	-/780	-/753	SMD 0.03(-0.10 to 0.15)	Not estimable	⊕⊕○○Low	IMPORTANT
VAS Scale	13	Randomizedtrials	Not serious	Seriousᶠ	Not serious	Not serious	None	-/945	-/957	SMD -0.23(-0.41 to -0.06)	Not estimable	⊕⊕⊕○Moderate	CRITICAL

CI: Confidence interval; RR: Risk ratio; SMD: Standardized mean difference.Explanations• a. Heterogeneity = 76%.• b. Heterogeneity = 50%.• c. Wide variation in confidence intervals across several time points.• d. Heterogeneity = 51%.• e. Several confidence intervals crossed the null effect.

## Data Availability

Not available.
